# Efficacy and safety of available treatments for visceral leishmaniasis in Brazil: A multicenter, randomized, open label trial

**DOI:** 10.1371/journal.pntd.0005706

**Published:** 2017-06-29

**Authors:** Gustavo Adolfo Sierra Romero, Dorcas Lamounier Costa, Carlos Henrique Nery Costa, Roque Pacheco de Almeida, Enaldo Viera de Melo, Sílvio Fernando Guimarães de Carvalho, Ana Rabello, Andréa Lucchesi de Carvalho, Anastácio de Queiroz Sousa, Robério Dias Leite, Simone Soares Lima, Thais Alves Amaral, Fabiana Piovesan Alves, Joelle Rode, the Collaborative LVBrasil Group

**Affiliations:** 1 Núcleo de Medicina Tropical, Universidade de Brasília, Brasília, Distrito Federal, Brasil; 2 Universidade Federal do Piauí, Hospital de Doenças Tropicais Natan Portela, Teresina, Piauí, Brasil; 3 Universidade Federal de Sergipe, Hospital Universitário, Aracaju, Sergipe, Brasil; 4 Departamento de Doenças Infecciosas, Hospital Universitário Clemente de Faria, Universidade Estadual de Montes Claros, Montes Claros, Minas Gerais, Brasil; 5 Laboratório de Pesquisa Clínica, e Políticas Públicas em Doenças Infecciosas e Parasitárias, Centro de Pesquisas René Rachou, Fundação Oswaldo Cruz, Belo Horizonte, Minas Gerais, Brasil; 6 Hospital Infantil João Paulo II, Fundação Hospitalar do Estado de Minas Gerais, Belo Horizonte, Minas Gerais, Brasil; 7 Universidade Federal do Ceará, Hospital São José de Doenças Infecciosas, Fortaleza, Ceará, Brasil; 8 Universidade Federal do Piauí – Hospital Infantil Lucídio Portela, Teresina, Piauí, Brasil; 9 Plataforma de Pesquisa Clínica, Vice-Presidência de Pesquisa e Laboratórios de Referência, Fundação Oswaldo Cruz, Rio de Janeiro, Rio de Janeiro, Brasil; 10 Drugs for Neglected Diseases *initiative* (DND*i*), Geneva, Switzerland; 11 Drugs for Neglected Diseases *initiative* (DND*i*), Rio de Janeiro, Rio de Janeiro, Brasil; RTI International, UNITED STATES

## Abstract

**Background:**

There is insufficient evidence to support visceral leishmaniasis (VL) treatment recommendations in Brazil and an urgent need to improve current treatments. Drug combinations may be an option.

**Methods:**

A multicenter, randomized, open label, controlled trial was conducted in five sites in Brazil to evaluate efficacy and safety of (i) amphotericin B deoxycholate (AmphoB) (1 mg/kg/day for 14 days), (ii) liposomal amphotericin B (LAMB) (3 mg/kg/day for 7 days) and (iii) a combination of LAMB (10 mg/kg single dose) plus meglumine antimoniate (MA) (20 mg Sb^+5^/kg/day for 10 days), compared to (iv) standard treatment with MA (20 mg Sb^+5^/kg/day for 20 days). Patients, aged 6 months to 50 years, with confirmed VL and without HIV infection were enrolled in the study. Primary efficacy endpoint was clinical cure at 6 months. A planned efficacy and safety interim analysis led to trial interruption.

**Results:**

378 patients were randomized to the four treatment arms**:** MA (n = 112), AmphoB (n = 45), LAMB (n = 109), or LAMB plus MA (n = 112). A high toxicity of AmphoB prompted an unplanned interim safety analysis and this treatment arm was dropped. Per intention-to-treat protocol final analyses of the remaining 332 patients show cure rates at 6 months of 77.5% for MA, 87.2% for LAMB, and 83.9% for LAMB plus MA, without statistically significant differences between the experimental arms and comparator (LAMB: 9.7%; CI95% -0.28 to 19.68, p = 0.06; LAMB plus MA: 6.4%; CI95% -3.93 to 16.73; p = 0.222). LAMB monotherapy was safer than MA regarding frequency of treatment-related adverse events (AE) (p = 0.045), proportion of patients presenting at least one severe AE (p = 0.029), and the proportion of AEs resulting in definitive treatment discontinuation (p = 0.003).

**Conclusions:**

Due to lower toxicity and acceptable efficacy, LAMB would be a more suitable first line treatment for VL than standard treatment. ClinicalTrials.gov identification number: NCT01310738.

**Trial registration:**

ClinicalTrials.gov NCT01310738

## Author summary

Visceral leishmaniasis remains a worldwide public health concern with high mortality even when proper treatment is instituted. There is a need to develop efficacious, safer and shorter treatment alternatives as the current options suffer from high toxicity and long treatment duration. Combination therapies emerge as an alternative, and WHO has encouraged the conduct of studies to evaluate drug combinations where evidence for current treatment regimens is not available. In Brazil, there is no local evidence to support the current treatment recommendations. Therefore, a clinical trial was conducted in five hospitals in Brazil to evaluate the efficacy and safety of the current available treatments—meglumine antimoniate, amphotericin B deoxycholate and liposomal amphotericin B—and of a combination of liposomal amphotericin B, single dose, and meglumine antimoniate, in a shorter administration regimen. Preliminary safety results led to the discontinuation of the amphotericin B deoxycholate treatment arm and a planned interim analysis resulted in the trial interruption. The final results, comparing liposomal amphotericin B and liposomal amphotericin B plus meglumine antimoniate to the standard meglumine antimoniate treatment, did not show a statistically significant difference in cure rates, though cure rate was higher in the liposomal amphotericin B group. Liposomal amphotericin B treatment showed a better safety profile compared to meglumine antimoniate. These results will support future changes in treatment protocols in Brazil and potentially in Latin America.

## Introduction

Visceral leishmaniasis (VL) is a public health problem in developing countries. The overall annual incidence is estimated at approximately 200,000 to 400,000 cases [1], with a case-fatality rate of 10% per year (*i*.*e*. 20,000 to 40,000 deaths per year) [2]. In Latin America, VL is a zoonosis caused by *L*. *infantum* with dogs being the main reservoir. More than 95% of VL reported cases in Latin America occur in Brazil, with more than 40% of those in children under 10 years of age [3,4]. In Brazil, the disease has spread from the rural North East region throughout the national territory over the past 30 years, causing epidemics in the fast-growing outskirts of many large and medium-sized cities [5]. Between 1990 and 2014, Brazil reported a total of 78,433 cases, with an average of 3,137 new cases per year [6]. Recent studies have demonstrated increasing case-fatality rates [7] and poor prognosis has been associated with the presence of jaundice, thrombocytopenia, hemorrhage, HIV coinfection, diarrhea, age <5 and age >40–50 years, severe neutropenia, dyspnea and bacterial infections [8]. In Brazil, the current first-line treatment for VL is meglumine antimoniate—MA (20 mg Sb^+5^/kg/day for 20 days), with amphotericin B deoxycholate—AmphoB (1 mg/kg/day for 14 days) or liposomal amphotericin B—LAMB (3 mg/kg/day for 7 days) being second-line treatments [5]. However, there is no local evidence on the efficacy and safety of these recommended treatment regimens [9]. The national treatment recommendations are based on an expert consensus based on data obtained from studies carried out in other VL-endemic geographies (e.g. Europe, Asia) and local medical practice. As recommended treatments present serious patient management limitations due to toxicity, a need for parenteral administration and hospitalization, the risk of resistance development, and cost, alternative drugs and treatment regimens for VL are needed. Although a series of new, highly active compounds have been identified, these are still in pre-clinical or earlier phases of development. In the short term, organizations such as the Drugs for Neglected Diseases Initiative (DND*i*) have adopted a strategy of investigating combination therapies in order to improve current treatment regimens. Clinical trials conducted on the Indian subcontinent and in Africa have shown the efficacy and safety of different treatment combinations: sodium stibogluconate (SSG) plus paromomycin (PM) in East Africa [10], and LAMB plus miltefosine, LAMB plus PM and miltefosine plus PM in India [11].

Based on this evidence, the WHO Expert Committee on the Control of Leishmaniasis reviewed VL treatment guidelines, and recommended the use of SSG plus PM for the treatment of VL in East Africa and drug combinations based on LAMB, miltefosine and PM on the Indian subcontinent. The committee also encouraged the conduct of studies to evaluate drug combinations in those endemic geographies where data on various treatment regimens were not available [12].

Given the potential drug susceptibility variations due to different *Leishmania* species in Latin America, evidence from other regions cannot be extrapolated to Brazil. In order to provide evidence for the rational use of VL drugs and treatment regimens, the Brazilian Ministry of Health requested and sponsored a clinical trial to assess the safety and efficacy of the three treatments recommended for VL in Brazil. The initial protocol was reinforced with a combination arm of LAMB single dose plus MA for ten days, suggested by DND*i* in 2009, after consultation with local experts, investigators and representatives of the National Control Program of the Ministry of Health. The objective of the trial was to assess the efficacy and safety of AmphoB, LAMB and a combination of LAMB plus MA, compared to the standard MA treatment.

## Methods

### Ethics statement

The study protocol and further amendments were approved by the institutional Ethics Committees of all the partner institutions: University of Brasilia, Brasilia; Federal University of Piauí, Teresina; Montes Claros State University, Minas Gerais; Pediatric Hospital Joao Paulo II—FHEMIG, Belo Horizonte, Minas Gerais; René Rachou Research Centre, FIOCRUZ, Minas Gerais; Federal University of Sergipe, Aracaju; Sao José Hospital of Infectious Diseases, Fortaleza, Ceará.

The study was conducted in accordance with the declaration of Helsinki, the International Committee on Harmonization guidelines for Good Clinical Practices (ICH—GCP) and all applicable local requirements for the conduct of research on human subjects. A written ‘Informed Consent Form’ (ICF) was obtained from all study participants before enrollment in the trial. Written informed consent was provided by the parents or legal representative of study participants under the age of 18 years and, additionally, minors aged between 12 and 17 years also provided a written assent form. The study was registered in ClinicalTrials.gov under the identification number NCT01310738.

### Study design

The study was designed as a multicenter, randomized, open label, controlled trial to evaluate the efficacy and safety of AmphoB, LAMB and the combination LAMB plus MA, compared to standard treatment with MA. The patient allocation ratio was 1:1:1:1.

### Study period

The study started in January 2011. In September 2012 an unplanned interim safety analysis was performed that led to the suspension of AmphoB arm. In April 2014, enrollment of the remaining three treatment arms was interrupted, in accordance with the Data Safety Monitoring Board (DSMB) recommendation, after a planned efficacy and safety interim analysis had been completed. The last 6 months patient follow-up evaluation was concluded in October 2014.

### Study population and sites

Participants were initially enrolled at four clinical trial sites: Federal University of Piauí, Teresina; Montes Claros State University, Montes Claros; Pediatric Hospital Joao Paulo II—FHEMIG, Belo Horizonte; and Federal University of Sergipe, Aracaju, with the inclusion of a fifth trial site in 2012, Sao José Hospital of Infectious Diseases, Fortaleza, Ceará.

Inclusion criteria were defined as male and female patients aged from > 6 months to < 50 years, with a confirmed diagnosis of VL defined as fever > 37.8°C for at least one week, associated with hepatomegaly or splenomegaly, and a positive result in at least one of the following laboratory diagnostic tests: direct microscopic examination of bone marrow (BM) aspirate or parasite culture, polymerase chain reaction (PCR) on BM aspirate or peripheral blood samples, or the rK39 immunochromatographic rapid test (Kalazar Detect; InBios International Inc., Seattle, USA; IT—LEISH, Bio-Rad, France). Exclusion criteria were pregnancy; HIV infection; underlying chronic or acute disease which would preclude evaluation of the participant’s response to study medication (e.g. diabetes, cardiac, renal, or hepatic impairment, schistosomiasis, malaria, tuberculosis); co-morbidities that may cause alterations of the immune system; any concomitant use of medication that may interfere with the therapeutic response or cause detrimental pharmacological interactions; previous treatment with any anti-leishmanial drugs in the past 6 months; drug abuse; previous history of hypersensitivity reaction to tested interventions; any condition that may hinder compliance with the planned scheduled visits; relapse cases; clinical signs of severe VL disease, such as generalized edema, jaundice, toxemic signs, and severe malnutrition; serum creatinine and bilirubin above the upper normal limit (UNL); prolonged prothrombin time with international normalized ratio (INR) > 2.0; or a platelet count < 20,000/mm³.

### Treatments

The four study arms were (i) MA (Glucantime—Sanofi), 20 mg Sb^+5^/kg/day intravenous (IV) for 20 days, with a maximum of 1,215 mg pentavalent antimony (Sb^+5^; three 5 mL vials) per day as the reference arm; (ii) AmphoB, (Anforicin B—Cristália), 1 mg/kg/day IV for 14 days; (iii) LAMB (AmBisome—Gilead), 3 mg/kg/day IV for 7 days; and (iv) a combination of LAMB, 10 mg/kg/day IV, single dose at first day, followed by MA, 20 mg Sb^+5^/kg/day IV, for 10 days, with a maximum of 1,215 mg of pentavalent antimony (three 5 mL vials) per day, as intervention arms. The choice of MA as the reference arm was based on the prevailing Brazilian VL treatment guidelines at the time when the study was started in 2011 [5], as well as data obtained from a clinical trial that had been concluded in 2009 [13]. AmphoB and LAMB regimens were included as these were routinely used in Brazil and had been included in 2011 into the new treatment guidelines to reduce the VL fatality rate [14]. The same regimen of AmphoB had also been used in the above-referenced clinical trial conducted in 2009 [13]. The selection of the combination therapy with LAMB single dose plus MA was based on the lack of local efficacy evidence for miltefosine and PM, and the satisfactory efficacy rates reported for MA, the first line treatment in the country. Since MA toxicity is dose and time-dependent, with occurrence of cardiotoxicity after three to four weeks of exposure [15], a reduction of treatment duration was expected to result in a significant improvement in safety. Although, based on the current medical practice in Brazil, a maximum daily dose of antimony was used to avoid toxicity, the upper limit dose would not have significantly impacted trial outcome due to the overwhelming proportion of pediatric participants in the study. The LAMB regimen was selected due to the limited data available for Brazil [16] and the encouraging results obtained by Sundar *et al*. (2010) in India [17]. We decided to use a combination regimen, considering the safety concerns with LAMB doses above 10 mg/kg, the dose used in India as a single-dose administration.

Participants needing rescue treatment were given: LAMB, 3 mg/kg/day IV for 7 days. All participants received supervised treatment.

After the first year of the study, the investigators raised a concern about the number of serious adverse events (SAEs) and treatment discontinuations observed in the AmphoB treatment arm. In order to protect the safety of VL patients, an unplanned safety interim analysis was performed in 2012 to assess the toxicity profile in 79 participants randomized to the four treatment arms (17 to MA, 20 to AmphoB, 22 to LAMB and 20 to LAMB plus MA). The analysis, which included adverse events (AE) related to study medication, showed that treatment with AmphoB was associated with statistically significant higher number of AEs, an increased risk of AEs that led to temporary suspension or treatment discontinuation, and AEs that required medical intervention. AmphoB treatment also showed a higher risk of SAEs when compared to LAMB monotherapy and in combination with MA. Consequently, the DSMB recommended dropping the AmphoB treatment arm from the trial.

### Sample size

As the trial objective was to compare three interventions with the standard treatment (MA), the sample size was determined to allow for detection, with 80% power (1- β) and 5% significance level (α = 0.05), of a at least 8% difference in efficacy of each treatment arm in relation to the reference arm. Assuming a 90% efficacy in the MA reference arm, and adjusting for a 10% loss to follow-up and 10% to maintain the power of comparison between the patient subgroups with parasitological diagnostic confirmation and only a positive rk39 diagnostic test (as defined in the inclusion criteria), it was estimated 165 participants would be required per treatment arm, for a total sample size of 660 participants.

After the AmphoB arm was stopped due to its higher toxicity, the trial’s sample size was reviewed. Adjustment for loss to follow-up was reduced to 5%, in accordance with the observations from the trial up to that moment. No more adjustment was made for those patients diagnosed with only a positive rK39 test, due to the wide validation of these tests by TDR/WHO on three continents, which allows for the inclusion of patients with clinical signs and a positive immunochromatographic test [18]. A final total sample size of 426 was calculated, with 142 participants per treatment arm.

### Randomization and blinding

Computer-generated randomization into the four treatment arms was done using the software *Quickcalcs—online calculators for scientists* (www.graphpad.com/quickcalcs/randomize1.cfm). Blocks of 28 treatment allocations were generated and placed in sealed, opaque envelopes that were sent to each clinical trial site, and only opened by trial clinicians or site investigators when a participant was included in the trial.

After the AmphoB arm was withdrawn, if an enrolled patient was allocated to this treatment arm, the subsequent envelope containing a new code was designated to the patient until they were allocated to any of the three remaining arms. This type of approach did not allow for blinding.

### Study procedures

Patients attending the clinical trial sites who were suspected of having VL or who were referred for VL diagnosis confirmation and treatment were submitted to clinical examination, laboratory tests, rk39 immunochromatographic rapid testing and BM aspiration, as per routine case management procedures at the sites. Patients with confirmed VL diagnosis by rK39 test and/or a positive microscopic examination of the BM aspirate were then invited to participate in the trial. Molecular diagnosis was performed in peripheral venous blood and bone marrow samples collected at baseline. Polymerase chain reaction (PCR) was applied in these samples targeting a 120bp minicircle conserved region of *Leishmania* DNA kinetoplast, as previously reported [19] and the assays were performed later on at the Laboratório de Pesquisa Clínica, Centro de Pesquisas René Rachou, FIOCRUZ, Minas Gerais and at the Leishmaniasis Laboratory of Natan Portella Institute in Teresina, Piauí.

During the informed consent process, they were informed of the trial objectives, procedures, interventions, risks and benefits. Participants then underwent thorough medical history and physical examination, including weight, height, vital signs, and spleen and liver size measurements. Complementary laboratory tests to confirm eligibility, and electrocardiogram (ECG) and chest radiography were performed. Participants who met the trial inclusion criteria were then randomly allocated to one of the four treatment arms.

After treatment was started at day (D) 0, participants were examined daily until they were discharged from hospital at the end of the interventions’ treatment period, and scheduled clinical and laboratory assessments were performed at D0, D3, D7, D14, D21, D30, D60, D90 and D180. ECG was performed at D7, D14 and D21 (and at additional time-points if indicated) and was evaluated by trial physicians. After a safety alert was raised by GlaxoSmithKline (GSK) regarding a potential risk of cardiac arrhythmia due to possible drug interaction between SSG and AmphoB [20], an additional safety assessment, consisting of potassium and serum creatinine measurement and ECG, was included at D1 for participants randomized to the LAMB plus MA combination arm.

### Endpoints

The primary efficacy endpoint was cure at 6 months follow-up, defined as complete remission of clinical signs and symptoms up to 3 months after the beginning of treatment, associated with normalization of hematological abnormalities observed at baseline, without evidence of relapse up to 6 months. Abnormal hemoglobin level at three and six months of follow up was taken into consideration when accompanied by other hematological abnormalities. Isolated anemia was not considered as a treatment failure criterion unless other hematological abnormalities or clinical signs and symptoms were present. Any abnormal white blood cells or platelet counting at D90 and D180 precluded clinicians of declaring the cure endpoint. Secondary efficacy endpoints were (i) clinical improvement at D30, defined as fever clearance, improvement or non-worsening of the hematological parameters, and spleen size reduction of any magnitude until D30; (ii) early therapeutic failure, defined as lack of clinical and/or laboratory responses described for ‘clinical improvement at D30’; (iii) therapeutic failure, defined as the absence of improvement or cure, or the need for early treatment interruption and rescue treatment; and (iv) relapse, defined as reappearance of symptoms after a period of improvement or complete remission, once specific VL treatment was completed, and occurring up to the 6 months follow-up visit. Other secondary endpoints included time to fever clearance from D0, mean proportion of spleen reduction at D30, and proportion of participants presenting splenomegaly at D60.

Safety endpoints included clinical and laboratory safety assessments of the different treatment arms as compared with standard treatment with MA. Safety was monitored daily during the hospitalization period and at the scheduled follow-up visits, through clinical examination and registry of participants´ complaints. Complete blood count, biochemistry tests and ECG, were performed according to scheduled evaluation times and whenever indicated by the investigator. AEs were recorded during the whole trial period and evaluated according to the Division of AIDS Table for Grading the Severity of Adult and Pediatric Adverse Events (DAIDS AE grading table), version 1.0, December 2004 [21]. Trial physicians performed AE causality assessment, and AEs were classified as related or not-related to the trial medications.

Participants withdrawn from the trial due to the occurrence of an AE received rescue treatment as described above, and were considered as an early treatment failure. Study coordination and Institutional Ethics Committees were notified of all SAEs, and an independent medical monitor (IMM) evaluated all SAE reports.

### Data handling and statistical analysis

Data were registered in a specific Case Report Form (CRF) from inpatients’ records and standard trial records were designed for the scheduled follow-up visits. Trial coordination designated monitors performed source document verification for all CRFs on site. Double data entry was performed in the Statistical Package for the Social Sciences—SPSS (IBM Statistics version 22).

The primary efficacy endpoint of cure at 6 months was analyzed as to the intention-to-treat (ITT) and per-protocol (PP) approaches. The ITT analysis included all participants randomized to the remaining three interventions, except for one participant randomized to the MA arm and withdrawn from the trial at D1 after a stool tested positive for schistosomiasis. Therefore, the ITT analysis included a total of 332 participants. The PP analysis excluded participants lost to follow-up and withdrawn from the trial because of the occurrence of AE/SAE.

Descriptive data were summarized using percentages, medians or means with their respective 25 and 75 percentiles or standard deviations as appropriate. Parametric (student T test) and non-parametric statistics (Mann Whitney U test) were used for comparison of continuous data, taking into consideration the normality of data distributions evaluated using the Kolomogorov-Smirnoff test and visual exploration of plotted data. Categorical comparisons were performed using the chi-square test or the exact Fisher test as appropriate. For all comparisons, 95% CI were calculated for the observed differences, as recommended by Altman *et al*. [22]. All comparisons were performed using SPSS software (IBM statistics version 22).

### Interim analyses

In Setember 2012, an unplanned interim safety analysis showed that treatment with AmphoB was associated with higher toxicity, resulting in the interruption of the allocation of participants to this intervention arm, following DSMB recommendations.

In 2014, a planned efficacy and safety interim analysis was performed after 50% of the recruited participants completed 6 months follow-up. The data showed cure rates as per ITT of 77.1% for MA standard arm treatment, 86.1% for LAMB monotherapy, and 83.1% for LAMB plus MA combination treatment. The ITT efficacy analysis comparing the intervention groups against the MA comparator did not show statistical significance (S1 Table). Considering the cure rate observed in both intervention arms and the cure rate observed in the MA comparator arm, efficacy assumptions would require adjustment leading to a significant increase of the sample size per treatment arm in order to maintain the 80% study power, hindering the conduct of the trial. Therefore, the DSMB recommended that patient recruitment be stopped and the trial was interrupted in April 2014, with a total of 378 patients randomized to four arms, who were followed until completion of the 6 months follow-up period. This publication presents the final efficacy and safety results of the trial.

## Results

### Participants

**Fig 1:**
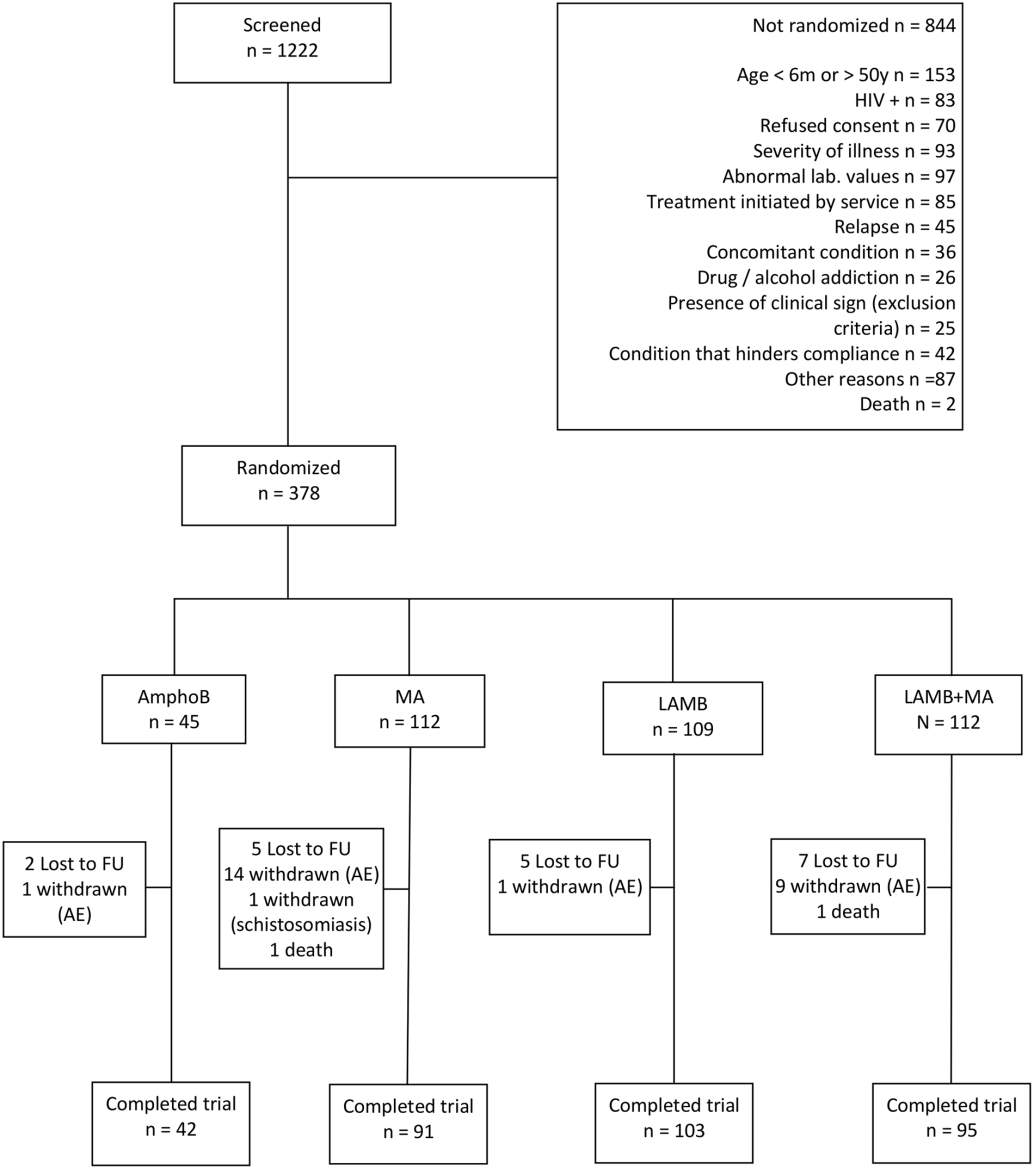
CONSORT patient flowchart. Flow diagram of the progress through the phases of the trial. AmphoB = amphotericin B deoxycholate; MA = meglumine antimoniate; LAMB = Liposomal amphotericin B.

As shown in Fig 1, a total of 1,222 patients with confirmed VL were screened, of which 378 (31%) were randomized to the four treatment arms. The main screening failure reasons were age outside the trial range (18.1%), abnormal laboratory tests as defined in the exclusion criteria (11.5%), severity of the illness (11.0%), specific treatment exposure before trial enrollment (10.1%), and a positive HIV test (9.8%). The sample was composed mainly by pediatric participants who were at least moderately ill.

### Protocol adherence

Among the 332 participants considered for these analyses, eighteen mild to moderate protocol violations were recorded during the study period. Three participants were randomized in spite of fulfilling exclusion criteria: (i) one in the LAMB group with bilirubin level above the UNL, and (ii) two in the MA group, of whom one had schistosomiasis diagnosed after randomization and of whom one was diagnosed with severe malnutrition. For two participants randomized to the MA group bilirubin or creatinine levels was not measured at baseline. Eight participants received one or two more doses of the treatment medication and two participants had treatment with MA with an interval between doses exceeding 72 hours. One participant did not receive the last MA dose and one did not receive the last two doses. Another patient randomized to the combination treatment arm received the single dose LAMB after completing MA administration. Though characterizing protocol deviations, all those participants were maintained in the PP analysis with their actual outcome registry as per evaluation at 6 months follow-up.

Nine participants had their six months follow-up visit after the allowed window (ranging from one day to 43 days) and were included in the ITT analysis as cured since if they were free of relapse after the allowed period, they were also free of relapse at 6 months. One patient had the six months visit 12 days before the allowed window, and as it was verified that he continued to be free of relapse after 6 months, he was also considered as cured for ITT analysis.

### Baseline characteristics

As shown in Table 1, treatment groups are comparable for baseline demographic, clinical and laboratory variables.

**Table 1:** Baseline demographic, clinical and laboratory characteristics of 332 participants effectively randomized to the three remaining arms of the trial.

Characteristics	MA n = 111	LAMB n = 109	LAMB + MA n = 112
**Demographic**			
Age (median, years)	4.77	4.36	3.36
Percentile 25 and 75	(2.05–10.37)	(2.13–10.27)	(1.84–9.19)
Interval	(0.61 to 47.61)	(0.56 to 48.64)	(0.49 to 48.83)
Male			
% (n)	58.6 (65/111)	54.1 (59/109)	52.7 (59/112)
Time of exposure in endemic area (median, years)	4.42 (n = 110)	3.96 (n = 108)	3.17 (n = 111)
Percentile 25 and 75	(1.83–9.94)	(1.88–8.92)	(1.75–8.00)
Interval	(0.25 to 42.92)	(0.42 to 48.67)	(0.50 to 44.08)
Time of illness evolution (median, months)	1.0	1.0 (n = 108)	0.80
Percentile 25 and 75	(0.5–2.0)	(0.5–2.0)	(0.5–2.0)
Interval	(0.16 to 30.0)	(0.23 to 13.0)	(0.16 to 12.00)
**Physical exam**			
Weight (median, kg)	16.7	15.2	15.0
Percentile 25 and 75	(12.0–28.5)	(11.6–29.4)	(10.8–30.6)
Interval	(2.0 to 84.7)	(6.5 to 76.0)	(5.8 to 79.0)
Splenomegaly			
% (n)	98.2 (109/111)	100 (109/109)	100 (112/112)
Spleen size below the left costal margin (median, cm)	6.5 (n = 109)	7.0 (n = 108)	7.0 (n = 107)
Percentile 25 and 75	(5.0–10.0)	(5.0–10.0)	(5.0–9.0)
Interval	(0.0 to 25.0)	(1.0 to 18.0)	(2.0 to 23.0)
Hepatomegaly			
% (n)	99.1 (110/111)	94.5 (103/109)	97.3 (109/112)
Liver size below the right costal margin (median, cm)	4.0	4.0 (n = 108)	4.0 (n = 109)
Percentile 25 and 75	(3.0–6.0)	(3.0–6.0)	(3.0–6.0)
Interval	(0.0 to 16.0)	(0.0 to 11.0)	(0.0 to 11.0)
Bleeding			
% (n)	2.7 (3/111)	2.8 (3/109)	0.9 (1/112)
Edema			
% (n)	9.9 (11/111)	6.4 (7/109)	7.1 (8/112)
**VL diagnosis tests**			
rK39 rapid test positivity			
% (n)	92.8 (103/111)	94.5 (103/109)	93.7 (104/111)
Positive direct examination for parasite in bone marrow smear			
% (n)	64.9 (61/94)	66.7 (60/90)	54.5 (54/99)
Positive culture of bone marrow aspirate			
% (n)	48.9 (45/92)	44.4 (40/90)	48.4 (45/93)
**Bacterial cultures**			
Positive blood culture			
% (n)	6.5 (7/108)	1.9 (2/107)	10.4 (11/106)
Positive urine culture			
% (n)	5.1 (5/98)	5.5 (5/91)	9.8 (9/92)
**Complete Blood Count**			
Leukocytes count/uL	2970	2880	2870
Percentile 25 and 75	(2000–4030)	(1990–4500)	(1880–3982)
Interval	(760 to 11300)	(880 to 12660)	(1000 to 9860)
Neutrophil count/uL	1056 (n = 109)	1071	995
Percentile 25 and 75	(616–1623)	(715–1616)	(683–1455)
Interval	(231 to 3130)	(209 to 4930)	(165 to 6802)
Hemoglobin g/dL	8.0	7.9	7.9
Percentile 25 and 75	(7.2–9.2)	(7.1–8.8)	(7.1–9.2)
Interval	(4.8 to 13.4)	(5.1 to 12.1)	(4.7 to 13.9)
Hematocrit %	25.7	25.1	25.0
Percentile 25 and 75	(23.2–28.9)	(22.6–28.0)	(22.8–28.5)
Interval	(16.5 to 38.4)	(15.6 to 38.6)	(14.3 to 45.7)
Platelet count /uL x10^3^	90	101	91
Percentile 25 and 75	(65.6–131)	(66.9–134)	(69–120)
Interval	(22 to 244)	(20 to 249)	(23 to 210)
**Clinical chemistry**			
Glucose mg/dL	82.0 (n = 107)	89.0 (n = 105)	86.0 (n = 103)
Percentile 25 and 75	(74.0–92.0)	(76.5–99.5)	(77.0–101.0)
Interval	(53.0 to 161.0)	(60.0 to 145.0)	(54.0 to 140.0)
Creatinine mg/dL	0.50 (n = 110)	0.50	0.50
Percentile 25 and 75	(0.9–0.62)	(0.40–0.62)	(0.40–0.60)
Interval	(0,10 to 1,00)	(0.10 to 1.50)	(0.10 to 1.20)
Sodium mEq/L	135	136 (n = 105)	136 (n = 110)
Percentile 25 and 75	(133 e 138)	(132 e 138)	(133 e 138)
Interval	(115 a 145)	(126 a 154)	(124 a 167)
Potassium mEq/L	4.2	4.3 (n = 104)	4.2 (n = 109)
Percentile 25 and 75	(3.9–4.5)	(3.9–4.6)	(3.9–4.5)
Interval	(3.2–5.4)	(2.9–5.3)	(2.7–5.1)
Magnesium mEq/L	2.1 (n = 104)	2.1 (n = 100)	2.1 (104)
Percentile 25 and 75	(1.9–2.3)	(1.9–2.3)	(1.8–2.3)
Interval	(1.5 to 2.8)	(1.3 to 2.7)	(0.8 to 2.9)
Total bilirubin mg/dL	0.50	0.50	0.50
Percentile 25 and 75	(0.40–0.60)	(0.35–0.70)	(0.40 to 0.70)
Interval	(0.19 to 1.50)	(0.10 to 2.05)	(0.10 to 1.30)
AST u/L	81 (n = 108)	76 (n = 108)	80
Percentile 25 and 75	(40–118)	(46–124)	(51–127)
Interval	(21–345)	(20–737)	(22–1371)
ALT u/L	40 (n = 109)	39 (n = 107)	38 (n = 111)
Percentile 25 and 75	(23–74)	(27–76)	(24–71)
Interval	(10 to 484)	(5 to 718)	(7 to 793)
Prothrombin time (s)	15.1	15.0	15.0
Percentile 25 and 75	(13.3–16.8)	(13.8–16.7)	(13.8–16.6)
Interval	(10.2 to 25.3)	(11.2 to 22.2)	(11.0 to 23.9)
INR	1.23	1.22	1.21
Percentile 25 and 75	(1.11–1.34)	(1.14–1.32)	(1.13–1.31)
Interval	(0.91 to 1.90)	(1.00 to 1.89)	(0.96 to 1.89)
Total Proteins g/dL	7.10 (n = 107)	7.10 (n = 108)	7.00 (n = 109)
Percentile 25 and 75	(6.40–7.90)	(6.34–8.05)	(6.46–7.73)
Interval	(4.80 to 10.90)	(4.22 to 10.98)	(5.20 to 12.03)
Albumin g/dL	2.97 (n = 107)	2.90	2.90 (n = 110)
Percentile 25 and 75	(2.60–3.45)	(2.60–3.30)	(2.52–3.20)
Interval	(1.70 to 4.10)	(1.60 to 4.82)	(1.87 to 4.30)
Globulin g/dL	4.22 (n = 106)	4.14 (n = 108)	4.00 (n = 109)
Percentile 25 and 75	(3.49–4.73)	(3.60–4.86)	(3.50 to 4.86)
Interval	(2.30 to 9.20)	(1.17 to 7.97)	(2.21 to 8.95)
A/G Ratio	0.70 (n = 106)	0.72 (n = 108)	0.70 (n = 109)
Percentile 25 and 75	(0.54–0.90)	(0.58–0.84)	(0.56–0.87)
Interval	(0.18 to 1.47)	(0.31 to 2.61)	(0.24 to 1.64)
Lipase u/L	43.5 (n = 108)	50.1 (n = 103)	50.0 (n = 106)
Percentile 25 and 75	(25.0–69.5)	(30.1–96.0)	(25.3–78.5)
Interval	(1.4 to 294.0)	(1.2 to 373.0)	(2.8 to 568.0)
Amylase u/L	40.0 (n = 109)	37.0 (n = 109)	41.0 (n = 111)
Percentile 25 and 75	(30.0–59.0)	(30.0–58.0)	(30.0–53.0)
Interval	(11.0 to 139.0)	(22.0 to 211.0)	(9.0 to 158.0)

Data are presented as n and % or median and interquartile range (percentile 25 and 75).

MA = meglumine antimoniate;

LAMB = liposomal amphotericin B;

LAMB+MA = treatment combination liposomal amphotericin B and meglumine antimoniate;

AST = aspartate aminotransferase;

ALT = alanine aminotransferase

VL diagnosis was confirmed by rk39 rapid test and by direct examination of bone marrow aspirate in 310 out of 331 (93.9%) and 175 out of 283 (61.8%) participants respectively. Bone marrow cultures were positive in 130 out of 275 participants (47.3%). PCR on peripheral blood and bone marrow samples showed positive results in 229 out of 240 participants (95.4%) and PCR in peripheral blood was positive for 213 out of 247 participants (86.2%).

### Efficacy results

As shown in Table 2, the efficacy at 6 months follow-up (primary endpoint) as per ITT analysis was 77.5% in the MA arm, 87.2% in the LAMB arm and 83.9% in the LAMB plus MA arm. All patients who were designated as initial failure and relapse, who were withdrawn due to the occurrence of AE/SAE, and who were lost to follow-up were considered as treatment failure in the ITT analysis (S2 Table). There was no statistically significant difference in the cure rates between each treatment intervention and the comparator.

**Table 2:** Treatment efficacy at six months follow-up as per ITT approach.

Treatment	% of participants cured (n/total)	% of participants not cured (n/total)	Difference in cure rate—% (95% CI)	p-value (χ^2^)
MA (Comparator)	77.5 (86/111)	22.5 (25/111)		
LAMB	87.2 (95/109)	12.8 (14/109)	9.7 (-0.28 to 19.68)	0.060^a^
LAMB + MA	83.9 (94/112)	16.1 (18/112)	6.4 (-3.93 to 16.73)	0.222^b^
Total	82.8 (275/332)	17.2 (57/332)		

MA = meglumine antimoniate;

LAMB = liposomal amphotericin B;

LAMB+MA = treatment combination liposomal amphotericin B and meglumine antimoniate;

^**a**^ P-value calculated for LAMB versus MA;

^**b**^ P-value calculated for LAMB+MA versus MA.

The per protocol analysis (Table 3) that excluded participants withdrawn from the study due to AE/SAE and patients lost to follow-up shows a cure rate at 6 months of 94.5% in the MA arm, 92.2% in the LAMB arm and 98.9% in the LAMB plus MA arm. Again, the difference between each treatment arm and the comparator was not statistically significant.

**Table 3:** Treatment efficacy at six months follow-up as PP approach.

Treatment	% of participants cured (n/total)	% of participants not cured (n/total)	Difference in cure rate—% (95% CI)	p-value (χ^2^)
MA (Comparator)	94.5 (86/91)	5.5 (5/91)		
LAMB	92.2 (95/103)	7.8 (8/103)	-2.3 (-9.23 to 4.60)	0.528^a^
LAMB+MA	98.9 (94/95)	1.1 (1/95)	4.4 (-0.73 to 9.53)	0.112^b^
Total	95.1 (275/289)	4.9 (14/289)		

MA = meglumine antimoniate;

LAMB = liposomal amphotericin;

LAMB+MA = treatment combination liposomal amphotericin B and meglumine antimoniate;

^a^ P-value calculated for LAMB versus MA;

^b^ P-value calculated using Fisher exact test for LAMB+MA versus MA

As shown in Table 4, the rate of early withdrawal due to the occurrence of AE/SAE was 13.5% for MA (15/111), 0.92% for LAMB (1/109) and 8.9% for the combination LAMB plus MA (10/112), with a statistically significant difference between the LAMB treatment arm and the comparator (P- value < 0.001).

**Table 4:** Early withdrawal rate due to the occurrence of AE/SAE during treatment as per ITT.

Treatment	Early withdrawal rate due to occurrence of AE/SAE—% (n/total)	Difference in early withdrawal rate—% (95% CI)	p-value (χ^2^)
MA (Comparator)	13.5 (15/111)		
LAMB	0.92 (1/109)	-12.6 (-19.18 to -5.97)	<0.001^a^
LAMB+MA	8.9 (10/112)	- 4.6 (-12,86 to 3,66)	0.278^b^
Total	7.8 (26/332)		

MA = meglumine antimoniate;

LAMB = liposomal amphotericin B;

LAMB+MA = treatment combination liposomal amphotericin B and meglumine antimoniate;

^a^ P-value calculated for LAMB versus MA;

^b^ P-value calculated for LAMB+MA versus MA

**Fig 2:**
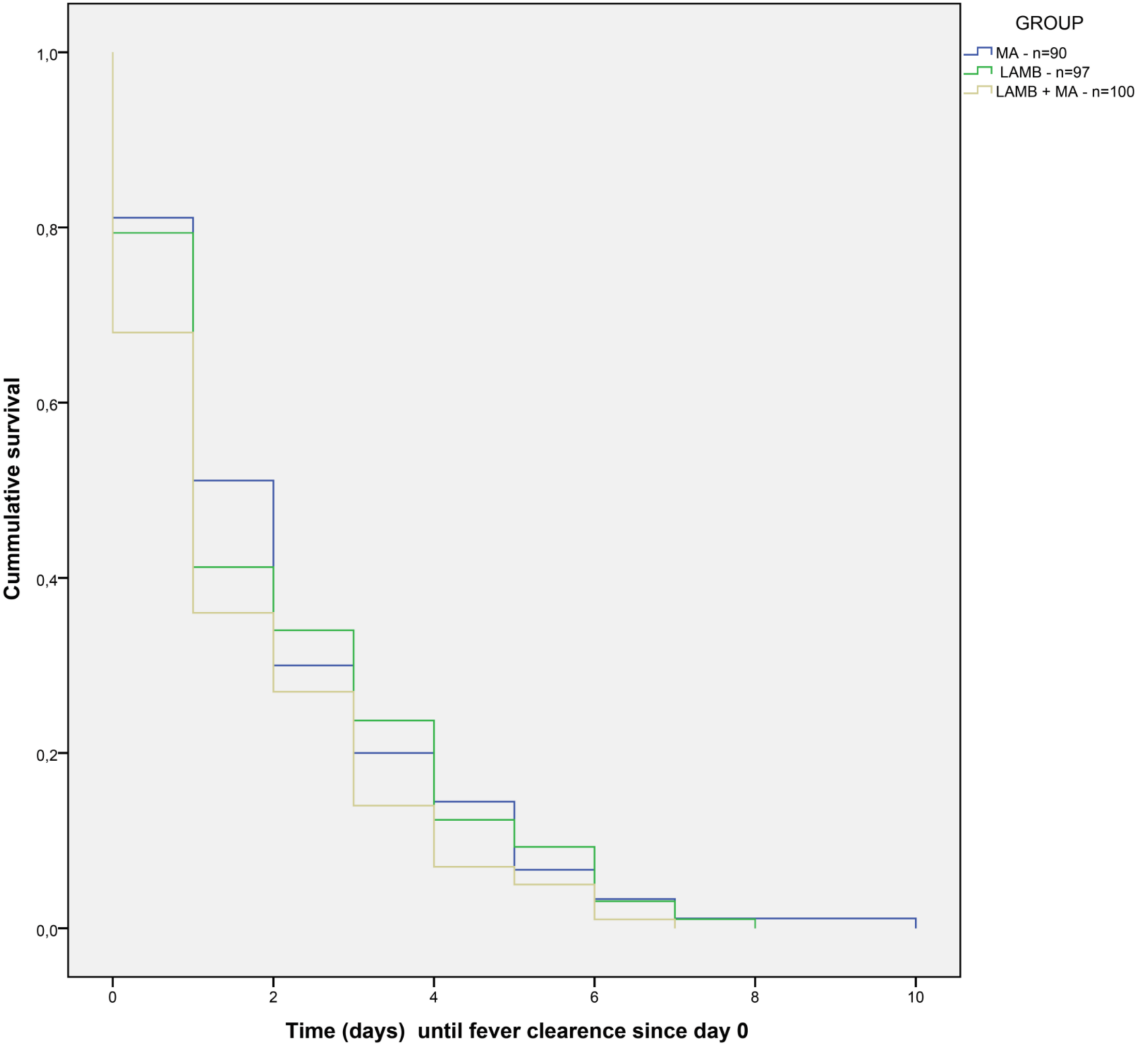
Survival time until fever clearance. Survival time until fever clearance per treatment arm by the Kaplan Meier method. MA = meglumine antimoniate; LAMB = Liposomal amphotericin B.

There was no statistically significant difference between each treatment arm and the comparator for the secondary endpoints of clinical improvement until D30 and relapse until D180 (S3 and S4 Tables). Survival analysis of time until fever clearance for 287 patients for whom data was available (Fig 2) did not show statistical differences between treatment arms (S5 Table). In all treatment arms there was a statistically significant reduction in spleen size between screening and D30, with a similar absolute difference in the mean reduction of spleen size of around 6 cm in all groups (S6 Table). At D60, there was no statistically significant difference between treatment intervention and the comparator in the proportion of participants still presenting splenomegaly (S7 Table). The data on spleen size reduction should be handled with caution because of limitations in spleen size measurement due to the inter and intra-observer variability, as there was no reliability assessment performed between study sites and between observers in the same trial site at the beginning of the trial.

### Safety results

Safety data were analyzed per participant and per event. The per participant analysis (Table 5) shows that 239 participants out of 332 (71.2%) presented at least one medication-related AE and that 66/332 (19.9%) presented at least one medication-related SAE. 61 participants out of the 66 who presented SAE (92.4%) presented one SAE, and 5 participants presented two SAEs (7.6%). There was no statistically significant difference in the percentage of participants presenting AE or SAE between each treatment arm and the comparator. The patient withdrawn in D1 due to a diagnosis of schistosomiasis did not present AE during his participation in the trial.

**Table 5:** Proportion of participants presenting at least one related AE, SAE and AE grade 3 and 4 per intervention arm.

	MA (comparator)	LAMB	LAMB+MA	Total
Number of participants	111	109	112	332
% of participants with AE (n)	74.8 (83)	65.1 (71)	75.9 (85)	71.2 (239)
Difference in % of participants with AE (95% CI)		-9.7 (-21.8 to 2.4)	1.1 (-10.2 a 14.4)	
p value (χ2)		0.119^b^	0.846^c^	
% of participants with SAE (n)^a^	30.6 (34)	27.5 (30)	37.5 (42)	31.9 (106)
% of participants with SAE related to study medication(n)	18.0 (20)	17.4 (19)	24.1 (27)	19.9 (66)
Difference in % of participants with SAE (95% CI)		- 0.6 (-10.7 to 9.5)	6.1 (-4.6 a 16.8)	
p value (χ2)		0.909^b^	0.265^c^	
% of participants with AE grade 3 or 4^d^ (n)	41.4 (46)	27.5 (30)	42.8 (48)	37.3 (124)
Difference in % of participants with AE grade 3 or 4^d^ (95% CI)		-13.9 (-26.3 to -1.5)	1.4 (-11.6 to 14.4)	
p value (χ2)		0.029^b^	0.83^c^	

MA = meglumine antimoniate;

LAMB = liposomal amphotericin B;

LAMB+MA = treatment combination liposomal amphotericin B and meglumine antimoniate;

^a^ All SAEs, including not related to study medication;

^b^ P-value calculated for LAMB versus MA;

^c^ P-value calculated for LAMB+MA versus MA;

^d^ Grade 3 = severe; Grade 4 = potentially life-threatening.

When analyzing AE severity, results show a statistically significant difference in the proportion of participants who presented at least one AE classified as severe (grade 3 and 4 according to DAIDS AE grading table [21] between the LAMB arm and comparator.

In the per event approach (Table 6), a total of 568 medication-related AEs were observed among the 239 patients who presented related AEs, of which 71 fulfilled the definition of SAE. Of these 568 medication-related AEs, 208 (36.6%) occurred in participants randomized to the MA arm, 143 (25.2%) in participants randomized to the LAMB arm and 217 (38.2%) in patients randomized to the LAMB plus MA arm. There was a smaller proportion of related AEs in the LAMB arm compared with MA comparator (p = 0.045). As far as SAEs are concerned, 21/71 (29.6%) occurred in the MA arm, 22/71 (31.0%) in the LAMB arm and 28/71 (39.4%) in the LAMB plus MA arm. There was no statistically significant difference between treatment arms and the comparator.

**Table 6:** Frequency of AE and SAE related to study medication, and temporary and definitive treatment suspension due to the occurrence of AE per intervention arm.

	MA (comparator)	LAMB	LAMB+MA	Total
Absolute number of AE	208	143	217	568
Median of AE per participant (P_25_-P_75_)	2 (1–4)	2 (1–3)	2 (1–3)	
p-value		0.045^a^	0.982^b^	
Absolute number of SAE	21	22	28	71
Median of SAE per participant (P_25_-P_75_)	0 (0–0)	0 (0–0)	0 (0–1)	
p-value		0.668^a^	0.303^b^	
No treatment suspension % (n)	88.5 (184)	97.2 (139)	89.4 (194)	91.0 (517)
Temporary treatment suspension % (n)	3.8 (8)	2.1 (3)	5.1 (11)	3.9 (22)
p-value		0.536^c^	0.542^e^	
Definitive treatment suspension % (n)	7.7 (16)	0.7 (1)	5.5 (12)	5.1 (29)
p-value		0.003^d^	0.369^e^	
Description of SAEs related to study medication				
Grade 3/4 increase of pancreatic and/or liver enzymes (%)	6 (28.6)	1 (4.5)	5 (17.8)	12 (16.9)
Cardiac toxicity (%)	4 (19.0)	-	4 (14.3)	8 (11.3)
Anemia (alone or associated with other abnormal hematological parameters) (%)	3 (14.3)	9 (40.9)	11 (39.3)	23 (32.4)
Neutropenia or thrombocytopenia (%)	3 (14.3)	3 (13.6)	3 (10.7)	9 (12.7)
Phlebitis / cellulitis	1 (4.8)	1 (4.5)	3 (10.7)	5 (7.0)
Fever	-	4 (18.2)	-	4 (5.6)
Allergic reaction	1 (4.8)	-	2 (7.1)	3 (4.2)
Cutaneous rash	1 (4.8)	-	-	1 (1.4)
Potassium reduction	-	1 (4.5)	-	1 (1.4)
Generalized edema	-	1 (4.5)	-	1 (1.4)
Convulsion	1 (4.8)	-	-	1 (1.4)
Sepsis	1 (4.8)	-	-	1 (1.4)
Diarrhea	-	1 (4.5)	-	1 (1.4)
Dyspnea	-	1 (4.5)	-	1 (1.4)

MA = meglumine antimoniate;

LAMB = liposomal amphotericin B;

LAMB+MA = treatment combination liposomal amphotericin B and meglumine antimoniate;

^a^ P-value calculated using Mann-Whitney test comparing LAMB versus MA;

^b^ P-value calculated using Mann-Whitney test comparing LAMB+MA versus MA;

^c^ p-value calculated using Fisher Exact test comparing LAMB versus MA;

^d^ p-value calculated using chi-square test comparing LAMB versus MA;

^e^ p-value calculated using chi-square test comparing LAMB+MA versus MA.

There were 51/568 (9%) of medication-related AEs resulting in temporary or definitive treatment suspension. For 22 episodes (3.9%), treatment interruption was transitory and for 29 occurrences (5.1%) treatment interruption was definitive. The proportion of medication-related AEs that resulted in definitive treatment suspension and need for rescue treatment in the LAMB intervention arm was shown to be significantly lower when compared with MA standard treatment (p = 0.003). There was no statistical difference in the proportion of definitive treatment suspensions between the LAMB plus MA arm and the comparator, nor in the proportions of transitory interruptions between each treatment arm and the comparator. In the MA and LAMB plus MA arms, the AES / SAEs that led to treatment discontinuation were mostly due to the increase of pancreatic and liver enzymes (10/16 or 62.5% and 6/12 or 50% respectively) and cardiac toxicity (2/16 or 12.5% and 4/12 or 30% respectively). One patient was discontinued in the LAMB arm due to a grade 4 increase in liver enzymes.

One of the 71 reported medication-related SAEs resulted in death. This participant was randomized to the MA treatment arm, presented respiratory and hemodynamic worsening on D3, was transferred to an intensive care unit and received rescue treatment with LAMB, but died of presumed sepsis on D11. Another patient randomized to the LAMB plus MA arm died on D2, due to a generalized infection by *Pseudomonas aeruginosa* confirmed at autopsy. This death was evaluated by the IMM as not being related to the study medication.

Treatment-related AEs reported in this trial are consistent with the expected toxicity profile of the drugs. S8 Table in supporting information shows treatment-related AEs per system organ class and intervention arm. Most of the treatment-related SAEs resolved during the hospitalization period. Participants presenting AE/SAE that led to their withdrawal from the trial and administration of rescue treatment were followed until complete resolution of the AE/SAE. All non-fatal medication-related SAEs that did not result in treatment interruption resolved within the 6 months follow-up period.

## Discussion

The present trial is the largest clinical trial ever conducted in the Americas to evaluate the efficacy and safety of the current recommended treatments for VL. The ITT analysis reported herein demonstrates that the overall cure rate obtained in the MA arm was lower than expected (77.5%). This precluded the possibility of proving a statistically significant difference compared with the LAMB arm (87.5%) in spite of the 9.7% crude efficacy difference between those groups favoring the LAMB arm. This is noteworthy: prior to the present trial, the general specialist opinion was that MA could achieve higher efficacy rates. The cure rate observed in the combination arm (LAMB plus MA) was also better than MA, but the crude efficacy difference was lower than for LAMB alone (6.4%).

The PP analysis, which focused on patients completing treatment, showed higher cure rates than those observed with the ITT approach. The PP analysis was essential to suggest that, once overcoming the important toxicity issues related to the administration of MA, patients exposed to MA in monotherapy or in combination present a tendency towards a higher cure rate. Those data reinforce the relevance of toxicity issues during VL treatment as important determinants of final outcomes and also call attention to the need for rescue treatment with the less toxic therapeutic option.

These final trial results are similar to those obtained in the planned efficacy and safety interim analysis that showed efficacy at the end of 6 months follow-up by ITT and PP analyses of 77.1% and 94.7% for MA, 86.1% and 91.2% for LAMB, and 83.1% and 98.3% for LAMB plus MA combination treatment, respectively, with no statistical significant differences in the cure rate between each treatment arm and the comparator for either set of analyses.

The absence of statistical significance between treatment arms and the comparator in the final analysis was expected. Thus, although a greater crude efficacy difference (9.7%) was observed than the 8% assumed a priori when designing the trial, the lower than expected MA efficacy (90%) meant that the study was not sufficiently powered.

Another study limitation is the fact that only non-severe cases of the disease and non-HIV infected participants were included, and therefore external validity of the trial results is only applicable to non-HIV infected patients who do not present severe illness.

Although efficacy evidence is scarce in Brazil and comes mostly from observational studies, high cure rates are reported in patients treated with MA. A retrospective study conducted at the Hospital of the University of Mato Grosso do Sul, Central-West Region of Brazil in 111 children treated with MA, showed a 96.9% efficacy rate in mild to moderate cases (93/96 patients who completed the standard 20 to 30 days treatment course) and over 64% in severely ill patients (7/11 patients). [23]. However, another retrospective observational study conducted in 89 patients with VL admitted in the Teaching Hospital Dr. Hélvio Auto in Maceió, State of Alagoas and treated with standard dose of MA for 1 to 40 days, for an average of 24.42 days, reported a 83.14% cure rate at 6 months for those 74 patients that had completed treatment; the study also showed that in 13.5% of the patients treatment had to be changed to AmphoB due to the occurrence of adverse reactions and three patients died (3.37%) [24]. Results of the present trial show an ITT efficacy for LAMB inferior to 90%, similar to previous studies: a clinical trial conducted simultaneously in India, Kenya and Brazil showed a 87% (13/15) efficacy of a LAMB regimen of 2 mg/kg/day on days 1 to 10 [16], while an efficacy superior to 90% has been observed in clinical trials conducted in other countries [25, 26]. Trials conducted in India in uncomplicated VL with different dose regimens showed LAMB efficacy greater than 90% [17, 27, 28, 29, 30]. Among these, two open-label, randomized trials using LAMB doses of 5 mg/kg reported efficacies of 91% at six and nine months (LAMB single dose) and 93% at six months (LAMB 5 mg/kg over 5 days) [27, 30].

In 2014, based on the efficacy results of single dose LAMB [17] and combination therapies of LAMB plus miltefosine, LAMB plus PM and miltefosine plus PM [11], the Indian National Road Map for Kala-Azar Elimination recommended the use of 10 mg/kg single dose LAMB as a first line treatment for VL patients in India, with PM and miltefosine as a second option at all levels [31].

In European countries, clinical trials have also shown efficacy of LAMB superior to 90% [32, 33, 34]. In Italy, an open label, dose-finding study in 88 patients showed 91% efficacy at 12 months with a LAMB regimen of 3 mg/kg on days 1 to 4 and 10 [33], though efficacy of 75% was found with the same total dose of 15 mg/kg in another dose-finding trial conducted in the same country in 106 children [32]. In both studies, total LAMB dose of 18 mg/kg (3 mg/kg on days 1 to 5 and 10) yielded a 98% efficacy rate at 12 months. LAMB total doses of 20 mg/kg and 21 mg/kg, as used in the present trial, have showed cure rates ranging from 90% to 100% [25, 32, 34].

Though the evidence is scarcer, LAMB does not appear as efficacious in Africa. While total doses of 14mg/kg and 10 mg/kg showed respectively 100% and 90% efficacy in a small number of patients in Kenya [16], recently, a multi-center, open-label, non-inferiority, randomized controlled trial conducted in 124 patients in Ethiopia and Sudan to compare the efficacy and safety of a single dose and multiple doses of LAMB showed efficacy at 6 months of 40% for a LAMB single dose of 7.5 mg/kg, 58% for a LAMB single dose of 10 mg/kg, and 85% for a LAMB regimen of 3 mg/kg on days 1–5, 14, and 21 [35]. The trial was terminated because of the low efficacy of the tested regimens.

Though results from other countries cannot be extrapolated to Brazil, the results of this trial suggest that there is room to improve LAMB efficacy, increasing the total dose by adjusting daily doses or length of treatment. Also, the hypothesis that parasite species (*L*. *donovani* in India and Africa, and *L*. *infantum* in Latin America and the Mediterranean region) and their susceptibility to drugs are determinants of the therapeutic outcomes observed across continents should be better explored in future trials. Furthermore, other alternative drug combinations could be explored as new chemical entities are progressing through development.

For the treatment of VL caused by *L*. *infantum*, WHO recommends, in order of preference, LAMB 3–5 mg/kg per daily dose given over a 3–6 days period, up to a total dose of 18–21 mg/kg [12]. PAHO incorporated this recommendation for the treatment of VL in the Americas, though the quality of evidence was evaluated as very low [36].

In Brazil, LAMB is indicated for patients aged < 1 year and > 50 years, severe illness based on severity score, renal, hepatic or cardiac insufficiency, HIV-coinfected patients, or other conditions leading to immunodeficiency, therapeutic failure to MA or other contraindications of use of MA [14, 37, 38]. The widening of LAMB indications in the country was motivated in part by the results of the unplanned safety analysis performed for the present trial, which showed increased toxicity in patients exposed to AmphoB.

Relapse was evaluated as a secondary endpoint; nonetheless, considering the scarcity of the event observed in the study (nine cases in 315 patients followed until six months), this issue remains to be addressed in future studies, principally in patients exposed to LAMB who presented the higher absolute number of relapse events in this trial (5/104) or when new interventions are assessed.

Regarding safety, there was no statistical significance between each treatment arm and the comparator in terms of the proportion of patients who presented medication-related AE/SAE, though the proportion of patients presenting AEs was lower in the LAMB intervention. However, patients exposed to LAMB presented a statistically significant lower frequency of AEs as compared to standard treatment. The proportion of AEs and SAEs among the total events observed in the trial was higher in the combination arm (217/568 or 38.2% and 28/71 or 39.4%, respectively) and SAE occurrence was mostly associated with MA toxicity.

A statistically significant difference between LAMB and the comparator MA arm was observed when comparing the proportion of patients presenting at least one severe AE (p = 0.029), as well as in the rate of early withdrawal due to the occurrence of AE/SAE (P < 0.001) and in the proportion of medication-related AEs that resulted in definitive treatment suspension and the need for rescue treatment (p = 0.003). Although there was no statistical difference in the proportion of transitory treatment suspensions between LAMB and comparator MA arm, participants exposed to LAMB presented a lower absolute number of temporary treatment interruptions (three *vs* eight in the MA arm and 11 in the LAMB plus MA arm).

The frequency of treatment interruption reported in the analysis of early withdrawal due to AE/SAE was lower than the frequency of definitive interruption caused by AE/SAE, because in the first analysis the participant was the analysis unit, while in the second, AE/SAE was the analysis unit; therefore, the occurrence of various concomitant AEs might have justified the treatment suspension in a single participant.

Overall, LAMB intervention showed an acceptable cure rate, and also showed a statistically significant better safety profile. This better tolerability profile, associated with a shorter administration time (seven days regimen vs 20 days for MA and 11 days for LAMB plus MA) and thus a reduced hospitalization period, suggest that LAMB monotherapy could be recommended as a more suitable first line treatment option for VL in Brazil and possibly in other Latin American countries where patients show a similar clinical profile.

The cost of medication is still a limiting factor for widening the use of LAMB. However, an agreement between the Brazilian Ministry of Health and the manufacturer of LAMB (Gilead) allows the drug’s purchase at a WHO negotiated price of 18 USD per 50 mg vial. Furthermore, the possibility of pooled purchase of the product through the PAHO Strategic Fund offers an opportunity for further price reduction. On the other hand, the higher costs of LAMB treatment should be put into context: toxicity observed with the current standard MA treatment results in costs associated with drug toxicity handling, longer hospitalization due to longer administration times, and ultimately, if treatment is interrupted, the need for rescue treatment. Such cost difference in treatment regimens should be evaluated in a cost-effectiveness study, which could provide additional evidence for the Ministry of Health in its potential revision of national VL treatment guidance.

### Conclusions

The results of the trial point towards a recommendation for the use of LAMB as the first line treatment for VL in Brazil and probably in other Latin American countries, due to its acceptable efficacy profile and its lower toxicity and shorter administration time as compared to MA and LAMB plus MA combination therapy.

## Supporting information

S1 Table Interim analysis efficacy at six months follow-up as per intention-to-treat approach. (DOCX)

S2 Table Characteristics of treatment failure per treatment group by intention-to-treat. (DOCX)

S3 Table Efficacy evaluated by clinical improvement at D30 of follow-up as per intention-to-treat. (DOCX)

S4 Table Efficacy evaluated by relapse rate at D180 as per intention-to-treat (complete case). (DOCX)

S5 Table Mean time until fever clearance per treatment arm by Kaplan Meier method. (DOCX)

S6 Table Mean spleen size reduction between screening and D30 by treatment arm. (DOCX)

S7 Table Proportion of participants presenting splenomegaly at D60 of follow-up. (DOCX)

S8 Table Frequency and proportion of treatment-related adverse events per system organ class and intervention arm. (DOCX)

S1 Consort Checklist (PDF)
