# Supplementary material for: Efficacy and safety of available treatments for visceral leishmaniasis in Brazil: A multicenter, randomized, open label trial
Source: PLoS Negl Trop Dis. 2017 Jun 29;11(6):e0005706. doi: 10.1371/journal.pntd.0005706 (PMC5507560; doi:10.1371/journal.pntd.0005706)
Supplement: S1 Table — (DOCX) [file pntd.0005706.s001.docx]

**S1 Table. Interim analysis efficacy at six months follow-up as per intention-to-treat approach**

| Treatment | % of participants cured (n/total) | % of participants not cured (n/total) | Difference in cure rate - % (95% CI) | p-value (χ^2^) |
| --- | --- | --- | --- | --- |
| MA (Comparator) | 77.1 (54/70) | 22.9 (16/70) |  |  |
| LAMB | 86.1 (62/72) | 13.9 (10/72) | 9.0 (-3.87 to 21.66) | 0.167**a** |
| LAMB + MA | 83.1 (59/71) | 16.9 (12/71) | 6.0 (-7.30 to 19.05) | 0.375**b** |
| Total | 82.1 (175/213) | 18.8 (40/213) |  |  |

MA = meglumine antimoniate; LAMB = liposomal amphotericin B; LAMB+MA = treatment combination liposomal amphotericin B and meglumine antimoniate; **a** P-value calculated for LAMB versus MA; **b** P-value calculated for LAMB+MA versus MA.
