# Supplementary material for: Efficacy and safety of available treatments for visceral leishmaniasis in Brazil: A multicenter, randomized, open label trial
Source: PLoS Negl Trop Dis. 2017 Jun 29;11(6):e0005706. doi: 10.1371/journal.pntd.0005706 (PMC5507560; doi:10.1371/journal.pntd.0005706)
Supplement: S2 Table — (DOCX) [file pntd.0005706.s002.docx]

**S2 Table. Characteristics of treatment failure per treatment group by intention-to-treat**

| Treatment | % of early therapeutic failure (n/total not cured) | % of withdrawal for AE/SAE (n/total not cured) | % of relapse (n/total) | % lost to follow-up (n/total not cured) |
| --- | --- | --- | --- | --- |
| MA (Comparator) | 8.0 (2/25) | 60.0 (15/25) | 12.0 (3/25) | 20.0 (5/25) |
| LAMB | 21.4 (3/14) | 7.1 (1/14) | 35.7 (5/14) | 35.7 (5/14) |
| LAMB+MA | 0.0 (0/18) | 55.6 (10/18) | 5.5 (1/18) | 39.8 (7/18) |
| Total | 8.8 (5/57) | 45.6 (26/57) | 15.8 (9/57) | 29.8 (17/57) |

MA = meglumine antimoniate; LAMB = liposomal amphotericin B; LAMB+MA = treatment combination liposomal amphotericin B and meglumine antimoniate.
