# Supplementary material for: Efficacy and safety of available treatments for visceral leishmaniasis in Brazil: A multicenter, randomized, open label trial
Source: PLoS Negl Trop Dis. 2017 Jun 29;11(6):e0005706. doi: 10.1371/journal.pntd.0005706 (PMC5507560; doi:10.1371/journal.pntd.0005706)
Supplement: S3 Table — (DOCX) [file pntd.0005706.s003.docx]

**S3 Table. Efficacy evaluated by clinical improvement at D30 of follow-up as per intention-to-treat**

| Treatment | % participants presenting clinical improvement (n/total) | % participants without improvement^a^ (n/total) | Difference in improvement rate versus comparator- % (95% CI) | P-value (χ^2^) |
| --- | --- | --- | --- | --- |
| MA (Comparator) | 82.9 (92/111) | 17.1 (19/111) |  |  |
| LAMB | 90.8 (99/109) | 9.2 (10/109) | 7.9 (-0.96 to 16.76) | 0.082^b^ |
| LAMB+MA | 89.3 (100/112) | 10.7 (12/112) | 6.4 (-2.65 to 15.45) | 0.167^c^ |
| Total | 87.7 (291/332) | 12.3 (41/332) |  |  |

MA = meglumine antimoniate; LAMB = liposomal amphotericin B; LAMB+MA = treatment combination liposomal amphotericin B and meglumine antimoniate;

^a^ Include therapeutic failure, early treatment interruption due to the occurrence of AE/SAE and lost to follow-up; ^b^ P-value calculated for LAMB versus MA; ^c^ P-value calculated for LAMB+MA versus MA.
