# Supplementary material for: Efficacy and safety of available treatments for visceral leishmaniasis in Brazil: A multicenter, randomized, open label trial
Source: PLoS Negl Trop Dis. 2017 Jun 29;11(6):e0005706. doi: 10.1371/journal.pntd.0005706 (PMC5507560; doi:10.1371/journal.pntd.0005706)
Supplement: S4 Table — (DOCX) [file pntd.0005706.s004.docx]

**S4 Table. Efficacy evaluated by relapse rate at D180 as per intention-to-treat (complete case)**

| Treatment | Relapse rate % (n/total) | Difference in relapse rate versus comparator - % (95% CI) | P-value (χ^2^) |
| --- | --- | --- | --- |
| MA (Comparator) | 3.3 (3/106) |  |  |
| LAMB | 4.8 (5/104) | 1.5 (-3.83 to 6.83) | 0.455^a^ |
| LAMB+MA | 1.0 (1/105) | -2.3 (-6.20 to 1.60) | 0.309^b^ |
| Total | 2.8 (9/315) |  |  |

MA = meglumine antimoniate; LAMB = liposomal amphotericin B; LAMB+MA = treatment combination liposomal amphotericin B and meglumine antimoniate; ^a^ P-value calculated for LAMB versus MA; ^b^ P-value calculated for LAMB+MA versus MA.
