# Supplementary material for: Efficacy and safety of available treatments for visceral leishmaniasis in Brazil: A multicenter, randomized, open label trial
Source: PLoS Negl Trop Dis. 2017 Jun 29;11(6):e0005706. doi: 10.1371/journal.pntd.0005706 (PMC5507560; doi:10.1371/journal.pntd.0005706)
Supplement: S5 Table — (DOCX) [file pntd.0005706.s005.docx]

**S5 Table. Mean time until fever clearance per treatment arm by Kaplan Meier method**

| Treatment | Mean (SD) | 95% CI | P-value (Log-rank) |
| --- | --- | --- | --- |
| MA (Comparator) (n = 90) | 2.100 (0.208) | (1.691 to 2.509) | <0.098 (Single comparison) |
| LAMB (n = 97) | 2.041 (0.202) | (1.645 to 2.437) |  |
| LAMB+MA (n = 100) | 1.580 (0.171) | (1.244 to 1.916) |  |

MA = meglumine antimoniate; LAMB = liposomal amphotericin B; LAMB+MA = treatment combination liposomal amphotericin B and meglumine antimoniate.
