# Supplementary material for: Efficacy and safety of available treatments for visceral leishmaniasis in Brazil: A multicenter, randomized, open label trial
Source: PLoS Negl Trop Dis. 2017 Jun 29;11(6):e0005706. doi: 10.1371/journal.pntd.0005706 (PMC5507560; doi:10.1371/journal.pntd.0005706)
Supplement: S6 Table — (DOCX) [file pntd.0005706.s006.docx]

**S6 Table. Mean spleen size reduction between screening and D30 by treatment arm**

| Treatment | Mean spleen size (cm) at screening (SD) | Mean spleen size (cm) at D30 (SD) | Difference in mean spleen size between screening and D30 - cm (95% CI) | P-value^a^ |
| --- | --- | --- | --- | --- |
| MA (Comparator) | 7.47 (4.16) | 1.90 (2.62) | 5.57 (4.95 to 6.20) | <0.001 |
| LAMB | 7.76 (3.38) | 1.50 (1.98) | 6.26 (5.67 to 6.86) | <0.001 |
| LAMB+MA | 7.25 (3.16) | 1.35 (2.21) | 5.90 (5.45 to 6.34) | <0.001 |

MA = meglumine antimoniate; LAMB = liposomal amphotericin B; LAMB+MA = treatment combination liposomal amphotericin B and meglumine antimoniate; ^a^ Paired Student t test.
