# Supplementary material for: Efficacy and safety of available treatments for visceral leishmaniasis in Brazil: A multicenter, randomized, open label trial
Source: PLoS Negl Trop Dis. 2017 Jun 29;11(6):e0005706. doi: 10.1371/journal.pntd.0005706 (PMC5507560; doi:10.1371/journal.pntd.0005706)
Supplement: S7 Table — (DOCX) [file pntd.0005706.s007.docx]

**S7 Table. Proportion of participants presenting splenomegaly at D60 of follow-up**

| Treatment | % of participants with splenomegaly | Difference in % of participants with splenomegaly versus comparator - % (95% CI) | P-value (χ^2^) |
| --- | --- | --- | --- |
| MA (Comparator) | 26.4 (24/91) |  |  |
| LAMB | 33.0 (31/94) | 6.6 (-6.53 to 19.72) | 0.326^a^ |
| LAMB +MA | 24.5 (24/98) | -1.9 (-14.33 to 10.53) | 0.766^b^ |
| Total | 27.9 (79/283) |  |  |

MA = meglumine antimoniate; LAMB = liposomal amphotericin B; LAMB+MA = treatment combination liposomal amphotericin B and meglumine antimoniate; ^a^ P-value calculated for LAMB versus MA; ^b^ P-value calculated for LAMB+MA versus MA.
