# Supplementary material for: Efficacy and safety of available treatments for visceral leishmaniasis in Brazil: A multicenter, randomized, open label trial
Source: PLoS Negl Trop Dis. 2017 Jun 29;11(6):e0005706. doi: 10.1371/journal.pntd.0005706 (PMC5507560; doi:10.1371/journal.pntd.0005706)
Supplement: S8 Table — (DOCX) [file pntd.0005706.s008.docx]

**S8 Table. Frequency and proportion of treatment-related adverse events per system organ class and intervention arm**

| Treatment | General/ systemic disorders n (%) | Administration site conditions n (%) | Cutaneous n (%) | Cardiovascular n (% ) | Gastrointestinal n (%) | Neurological n (%) | Respiratory n (%) | Genitourinary n (%) | Laboratory hematology n (%) | Laboratory biochemistry n (%) | Laboratory Urinalysis n (%) | Hematologic conditions n (%) | Bacterial infection n (%) | Total n (%) |
| --- | --- | --- | --- | --- | --- | --- | --- | --- | --- | --- | --- | --- | --- | --- |
| MA | 33 | 12 | 12 | 17 | 54 | 9 | 3 | 0 | 21 | 44 | 0 | 2 | 1 | 208 |
|  | (15.9) | (5.8) | (5.8) | (8.2) | (26.0) | (4.3) | (1.4) | (0.0) | (10.1) | (21.2) | (0.0) | (1.0) | (0.5) | (100.0) |
| LAMB | 34 | 4 | 5 | 4 | 28 | 2 | 5 | 1 | 24 | 32 | 1 | 3 | 0 | 143 |
|  | (23.8) | (2.8) | (3.5) | (2.8) | (19.6) | (1.4) | (3.5) | (0.7) | (16.8) | (22.4) | (0.7) | (2.1) | (0.0) | (100.0) |
| LAMB + MA | 44 | 9 | 9 | 15 | 34 | 3 | 2 | 2 | 34 | 58 | 0 | 7 | 0 | 217 |
|  | (20.3) | (4.1) | (4.1) | (6.9) | (15.7) | (1.4) | (0.9) | (0.9) | (15.7) | (26.7) | (0.0) | (3.2) | (0.0) | (100.0) |
| Total | 111 | 25 | 26 | 36 | 116 | 14 | 10 | 3 | 79 | 134 | 1 | 12 | 1 | 568 |
|  | (19.5) | (4.4) | (4.6) | (6.3) | (20.4) | (2.5) | (1.8) | (0.5) | (13.9) | (23.6) | (0.2) | (2.1) | (0.2) | (100.0) |

MA = meglumine antimoniate; LAMB = liposomal amphotericin B; LAMB+MA = treatment combination liposomal amphotericin B and meglumine antimoniate.
